# Glucolipid metabolism improvement in impaired glucose tolerance subjects consuming a Quinoa-based diet: a randomized parallel clinical trial

**DOI:** 10.3389/fphys.2023.1179587

**Published:** 2023-07-05

**Authors:** Hongli Zeng, Xiangsheng Cai, Zhenyang Qiu, Yuchan Liang, Lu Huang

**Affiliations:** Guangzhou Eleventh People’s Hospital, Guangzhou Cadre Health Management Center, Guangzhou, China

**Keywords:** quinoa, impaired glucose tolerance, blood glucose, blood lipid, influence

## Abstract

**Purpose:** To investigate the effects of quinoa on glucose and lipid metabolism, and the prognosis in people with impaired glucose tolerance.

**Methods:** One hundred and thirty-eight patients diagnosed with impaired glucose tolerance following a glucose tolerance test in Guangzhou Cadre Health Management Center were selected and randomly divided into quinoa intervention and control groups, according to the digital table method. After 1 year of follow-up, the differences in blood glucose, blood lipid, glycosylated hemoglobin and other indicators were compared. The disease prognosis between the two groups was also compared.

**Results:** The 2 h postprandial blood glucose, glycosylated hemoglobin, insulin resistance index, total cholesterol, low-density lipoprotein cholesterol, body mass index, waist circumference, systolic and diastolic blood pressure after intervention in the quinoa group were significantly lower than before intervention. In contrast, high-density lipoprotein cholesterol was higher than before intervention and is statistically significant (*p* < 0.05). After 1 year of follow-up, the control group’s glycosylated hemoglobin and body mass index are higher than before intervention, and are statistically significant (*p* < 0.05). The 2 h postprandial blood glucose, glycosylated hemoglobin, insulin resistance index, body mass index, and mean diastolic blood pressure in the quinoa group are statistically significantly lower than in the control group, while high-density lipoprotein cholesterol is higher (*p* < 0.05). The rate of conversion to diabetes for participants in the quinoa group (7.8%) is statistically significantly lower than in the control group (20.3%) (χ2 = 12.760, *p* = 0.002). Logistic regression analysis showed that quinoa consumption is a protective factor against delaying the progression of diabetes (*p* < 0.05).

**Conclusion:** Adding quinoa to staple food intake can reduce postprandial blood glucose, and improve lipid metabolism and insulin resistance, delaying the progression of diabetes in people with impaired glucose tolerance.

## Introduction

Pre-diabetes is a hyperglycemic state between normal blood glucose and diabetes (Association, 2020), including impaired fasting blood glucose (IFG) and/or impaired glucose tolerance (IGT), which is an important stage in the occurrence and development of diabetes. Previous studies have shown that pre-diabetes (Al Rifai and Shapiro, 2020)is associated with the risk of all-cause mortality, cardiovascular and cerebrovascular diseases, and heart failure. Pre-diabetes is reversible, and lifestyle intervention can delay the progression from pre-diabetes to diabetes (Khan et al., 2019). Studies have shown that Asia’s pre-diabetic population predominantly has impaired glucose tolerance (Yang W. et al., 2010).

Quinoa is a fake grain rich in high-quality protein, dietary fiber and trace elements (Vega-Gálvez et al., 2010). Quinoa seeds have recently gained attention due to their high-quality protein and extensive amino acid spectrum (Dakhili et al., 2019). Previous animal experiments have shown that the leachate of quinoa can significantly reduce the blood glucose levels of obese and hyperglycemic mice (Graf et al., 2014). Still, there are few relevant studies on the intervention of quinoa in the diet of people with impaired glucose tolerance. Only two studies on the influence of quinoa in people with pre-diabetes have been reported. (Díaz-Rizzolo et al., 2022) conducted an 8-week nutritional intervention (self-pairing) in nine pre-diabetic patients aged ≥65 years. The participants had 4 weeks on a conventional diet (RD) and 4 weeks on a quinoa diet (QD). Body weight, body mass index, and waist circumference decreased during the quinoa diet compared to the conventional diet. Additionally, postprandial blood sugar decreased during the quinoa diet. A second study (Abellán Ruiz et al., 2017), a randomized, placebo-controlled, double-blind study of 30 pre-diabetic subjects, showed that processed quinoa consumption over 28 days reduced body mass index and glycosylated hemoglobin levels and increased satiation in pre-diabetic patients without significantly affecting fasting blood glucose levels. The intervention duration of the two studies was short, with few subjects. The current study assessed changes in blood glucose, blood lipids, body mass index, waist circumference, blood pressure and disease prognosis in participants with impaired glucose tolerance after 1 year of intervention with a quinoa-modified diet. The influence of lifestyle on diabetes between the quinoa and control groups is also considered to ascertain dietary guidance for early reversal or delay of diabetes in people with impaired glucose tolerance.

## Participants and methods

**Materials:** The quinoa used in this study was white quinoa purchased from Anhui Yanzhifang Food Co., LTD., and produced in Qinghai, China. Cooking methods are steaming the quinoa alone or cooking it with other staple foods. Every 100 g quinoa rice contains 1573 KJ (19%) of energy, 12.7 g (21%) of protein, 5.9 g (10%) of fat, 67.0 g (22%) of carbohydrate, 9 mg (0%) of sodium, and 12.9 g (12.9%) of dietary fiber. The content of phytochemicals in quinoa was that the total polyphenol content was 781 mg gallic acid equivalent/100 g, the total flavonoid content was 321 mg rutin/100 g, and the saponin content was 170 mg oleanolic acid/100 g. Each bag of quinoa was 1000g, and the quinoa group was supplied with 3000 g of quinoa every month, asking them to replace the same amount of the refined rice or white flour products with 100 g of quinoa daily.

Subjects: One hundred and thirty-eight patients diagnosed with impaired glucose tolerance were selected who underwent oral glucose tolerance tests (OGTT) in Guangzhou Cadre Health Management Center between May to July 2021. Inclusion criteria: (1) people with reduced glucose tolerance (Association, 2020) (7.8≤ 2 h blood glucose after glucose load <11.1 mmol/L and fasting blood glucose <7.0 mmol/L); (2) Informed consent and voluntary participation in this study. Exclusion criteria: (1) patients with diagnosed diabetes; (2) People with other underlying severe diseases; (3) in acute infection, trauma or other stressful situations; (4) People with various mental illnesses, tumors, and pregnant women. This study was approved by the Ethics Committee of Guangzhou Cadre Health Management Center (Date 2021-07-19/No. k2022-12), and the patients signed an informed consent form.

In this study, the selected population is the group that underwent physical examination in our center every year, establishing a long-term connection with good compliance. This study does not require much intervention in the subjects’ daily life, does not require strict diet and exercise control, and does not violate traditional Chinese eating habits. The quinoa group needs to: (1) replace at least half of the refined rice or white flour products with quinoa as the daily staple food. (2) Fasting and postprandial blood glucose were measured once a week. (3) Keep in touch via wechat daily, take photos of food and upload them to log in. The control group received no particular intervention. During the study, those who did not clock in for more than 1 week were reminded, and those who did not clock in for more than 1 month were removed from the study.

**Sample size calculation**: Based on the estimated sample size of the disease incidence rate, the incidence rate progressing to diabetes of the quinoa group was assumed as 5%, and that of the control group was 20%. The sample size of each group was 73 through the PASS software, and the actual sample size of each group was 69.

**FIGURE 1:**
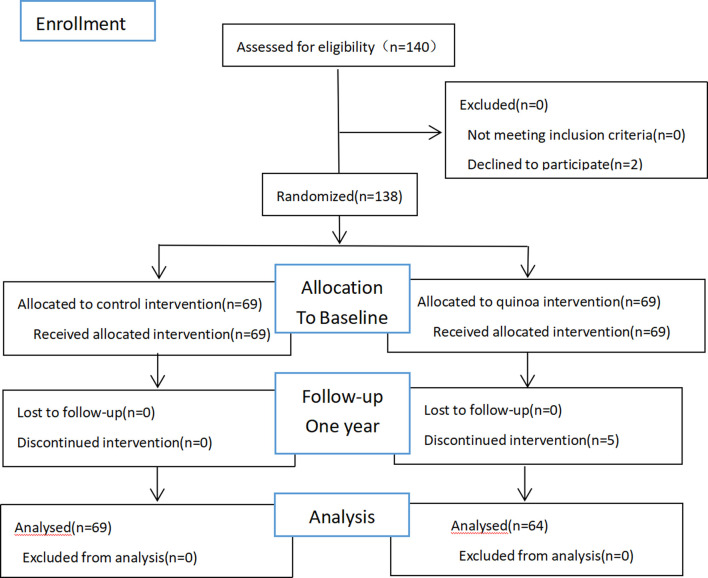
Allocation of the subjects of the quinoa study.

**Methods** Before enrollment, all subjects were surveyed with a lifestyle questionnaire. Trained staff guided participants to fill in, collect and document the data from the lifestyle questionnaire. The life questionnaire mainly includes questions such as a family history of diabetes, enjoying desserts or snacks, and sugary drink intake. The history of taking antihypertensive drugs, anti-lipid medication, cardiovascular and cerebrovascular proprietary Chinese medicines and healthcare products were collected. Oral glucose tolerance tests were performed before and after the study. According to the numerical table method, all participants were randomly divided into two groups, the quinoa intervention and control groups, with 69 participants in each group. In this experiment, the diet of the two groups was evaluated using wechat punch. A *per capita* intake of about 200 g of staple food per day was proposed. In the quinoa intervention group, 100 g of quinoa was used to replace the same amount of the refined rice or white flour products in the daily staple food for 1 year. The control group did not add staple food interventions, and they were suggested to avoid quinoa for 1 year. The two groups set up a wechat group, uploaded food photos and punched in daily, while the health manager collected feedback from the researchers, assessed dietary adherence and offered support. The participants in the study were educated about diabetes health knowledge to improve their understanding of the importance of lifestyle intervention in diabetes. In the quinoa intervention group, five patients dropped out of the study during follow-up. Sixty-four participants completed the study, including 30 males and 34 females, aged 39-68 years, with an average age of (56.83 ± 5.69) years. The control group has 69 subjects, including 35 males and 34 females, aged 45-76 years old, with an average age of (56.28 ± 6.48). There were no significant differences in age, gender, fasting blood glucose, 2 h postprandial blood glucose and Glycated hemoglobin between the two groups (*p* > 0.05) at the start of the study, indicating comparability, as shown in Table 1. Pre-intervention lifestyle characteristics between the two groups were statistically analyzed, and no differences were found. A Consort diagram is shown in Figure 1.

**TABLE 1:** The baseline information of study subjects before intervention.

Group	Count	age	Gender (male/female)	BMI	FBG	2hPG	HbA1c	HOMA-IR
Quinoa	64	56.83 ± 5.69	30/34	24.30 ± 2.50	5.99 ± 0.78	9.21 ± 0.94	5.93 ± 0.47	2.81 ± 1.26
Control	69	56.28 ± 6.48	35/34	24.39 ± 3.10	6.01 ± 0.59	9.16 ± 0.96	5.92 ± 0.43	2.75 ± 1.13
t/χ2		0.521	0.197	−0.196	−0.195	0.346	0.136	0.286
p		0.603	0.657	0.845	0.846	0.730	0.892	0.775

**Observation indicators:** Fasting blood glucose (FBG), 2 h postprandial blood glucose (2 hPG), glycosylated hemoglobin (HbA1c), fasting insulin levels, total cholesterol (TC), triglyceride (TG), low-density lipoprotein cholesterol (LDL-C) and high-density lipoprotein (HDL-C) were used to evaluate the participants over the 1 year. Height, weight, waist circumference (WC), systolic blood pressure (SBP) and diastolic blood pressure (DBP) of the two groups were measured. Calculated body mass index (BMI) = weight (Kg)/height 2 (m2); Insulin Resistance Index (HOMA-IR) = fasting glucose level x fasting insulin level/22.5. The correlation between diabetes and different lifestyles was analyzed 1 year later.

**Statistical methods:** SPSS 26.0 software was used for statistical analysis, and the measurement data were expressed as mean ± standard deviation (χ- ± s). Independent sample t-test was used for inter-group comparison, and paired t-test was used for intra-group comparison. The chi-square test was used for counting data, and the Rank sum test was used to compare rank variables between groups. The influencing factors of diabetes were analyzed using binary Logistic regression. *p* < 0.05 meant the difference was statistically significant.

## Results

### Changes in glucose metabolism indexes

The changes in glucose metabolism indexes are shown in Table 2. There are no significant differences in fasting blood glucose, 2 h postprandial blood glucose, glycosylated hemoglobin and insulin resistance index between the quinoa and control groups before dietary intervention (*p* > 0.05). After the dietary intervention with quinoa, the 2 h postprandial blood glucose, glycosylated hemoglobin and insulin resistance index in the quinoa group are significantly lower than those in the control group, with statistical significance (*p* < 0.05). Following the intervention, compared with the same group, it was found that the 2 h postprandial blood glucose, glycosylated hemoglobin and insulin resistance index in the quinoa group were lower than before quinoa was introduced. In contrast, the glycosylated hemoglobin in the control group is higher than before intervention, with statistical significance (*p* < 0.05).

**TABLE 2:** Changes of FBG, 2hPG, HbA1c and HOMA-IR in the two groups before and after dietary intervention with quinoa.

Group	FBG		2hPG		HbA1c		HOMA-IR	
	Pre-intervention	Post-intervention	Pre-intervention	Post-intervention	Pre-intervention	Post-intervention	Pre-intervention	Post-intervention
Quinoa	5.99 ± 0.78	5.92 ± 0.70	9.21 ± 0.94	8.65 ± 1.35*	5.93 ± 0.47	5.79 ± 0.52*	2.81 ± 1.26	2.32 ± 1.10*
Control	6.01 ± 0.59	6.13 ± 0.57	9.16 ± 0.96	9.57 ± 1.42	5.92 ± 0.43	6.03 ± 0.40*	2.75 ± 1.13	2.76 ± 1.21
t	−0.195	−1.917	0.346	−3.830	0.136	−3.077	0.286	−2.243
*p*	0.846	0.057	0.730	<0.001	0.892	0.003	0.775	0.027

Compared with the same group before intervention, *p < 0.05.

### Changes in lipid metabolism indexes

The changes in lipid metabolism indexes are shown in Table 3. Before the quinoa intervention, the mean value of total cholesterol in the quinoa group was significantly higher than in the control group (*p* < 0.05). Still, there was no statistically significant difference between triglycerides, low-density lipoprotein cholesterol and high-density lipoprotein cholesterol (*p* > 0.05). Following quinoa intervention, total cholesterol and high-density lipoprotein cholesterol levels in the quinoa group are significantly higher than in the control group (*p* < 0.05). For participants within the quinoa group, the total cholesterol and low-density lipoprotein cholesterol are significantly lower than before adding quinoa to their diet, while the high-density lipoprotein cholesterol is significantly higher than before intervention (*p* < 0.05). There are no significant differences in the control group before and after the 1-year study (*p* > 0.05).

**TABLE 3:** Changes of TC, TG, LDL-C and HDL-C in the two groups before and after intervention.

Group	TC		TG		LDL-C		HDL-C	
	Pre-intervention	Post-intervention	Pre-intervention	Post-intervention	Pre-intervention	Post-intervention	Pre-intervention	Post-intervention
Quinoa	5.71 ± 1.02	5.51 ± 0.98*	1.61 ± 1.17	1.55 ± 1.07	3.31 ± 0.85	3.15 ± 0.82*	1.40 ± 0.34	1.60 ± 0.32*
Control	5.35 ± 0.87	5.18 ± 0.90	1.88 ± 2.44	1.48 ± 0.95	3.38 ± 0.85	3.31 ± 0.71	1.34 ± 0.26	1.34 ± 0.36
t	2.238	1.984	−0.806	0.442	−0.446	−1.205	1.188	4.858
*p*	0.027	0.049	0.422	0.660	0.656	0.230	0.237	<0.001

Compared with the same group before intervention, *p < 0.05.

### Changes in other biomarkers and indexes

The changes in other indicators are shown in Table 4. Before the quinoa dietary intervention, no significant differences in body mass index, waist circumference, and systolic and diastolic blood pressure were noted between the quinoa and control groups (*p* > 0.05). Following the quinoa intervention, the participant’s body mass index and diastolic blood pressure are significantly lower than those of the control group (*p* < 0.05). Additionally, within the quinoa group, body mass index, waist circumference, and systolic and diastolic blood pressure are significantly lower than before the dietary intervention (*p* < 0.05). In the control group, the body mass index after the 1-year study is significantly higher than at the start of the study (*p* < 0.05), while there was no statistically significant difference in other indicators (*p* > 0.05).

**TABLE 4:** Changes in BMI, WC, SBP and DBP in the two groups before and after 1 year.

Group	BMI		WC		SBP		DBP	
	Pre-intervention	Post-intervention	Pre-intervention	Post-intervention	Pre-intervention	Post-intervention	Pre-intervention	Post-intervention
Quinoa	24.30 ± 2.50	23.71 ± 2.11*	83.48 ± 7.14	82.36 ± 6.41*	123.63 ± 13.44	121.95 ± 12.41*	72.05 ± 9.37	70.47 ± 9.43*
Control	24.39 ± 3.10	24.70 ± 3.07*	84.71 ± 9.42	84.55 ± 9.01	124.81 ± 16.82	124.25 ± 13.8	74.77 ± 11.25	73.96 ± 10.50
t	−0.196	−2.180	−0.841	−1.625	−0.447	−1.005	−1.509	−2.010
*p*	0.845	0.031	0.402	0.107	0.655	0.317	0.134	0.047

Compared with the same group before intervention, *p < 0.05.

### Comparison of prognosis between the two groups following quinoa intervention

**FIGURE 2:**
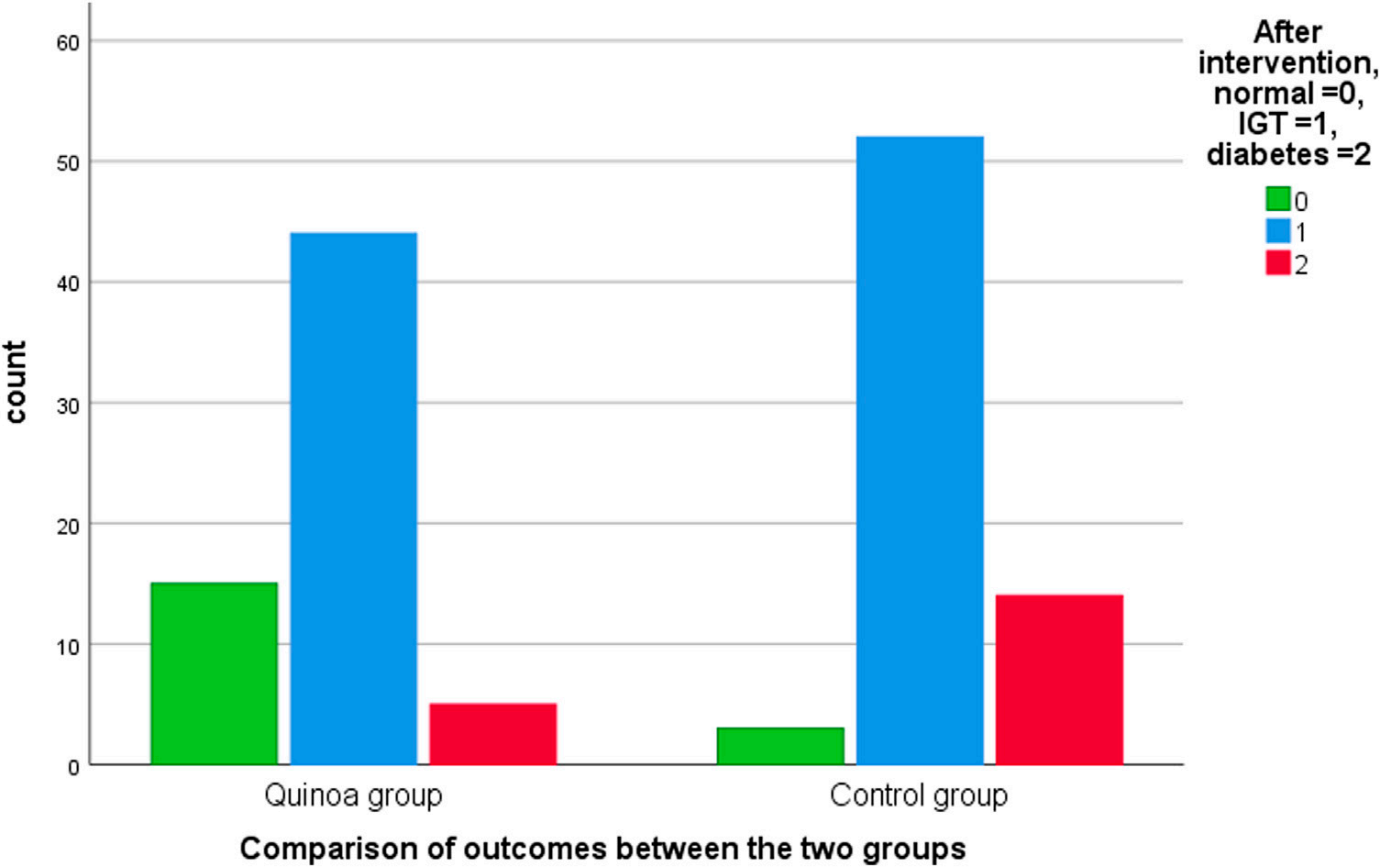
Comparison of outcomes between the two groups after 1 year.

The prognosis comparison between the two groups after the dietary intervention is shown in Figure 2. After 1 year of study, 5 participants (7.8%) progressed to diabetes, 44 patients (68.8%) stabilized to impaired glucose tolerance, and 15 cases (23.4%) returned to normal glucose tolerance in the quinoa group. In contrast, 14 patients (20.3%) in the control group progressed to diabetes, 52 cases (75.4%) stabilized as impaired glucose tolerance, and 3 participants (4.3%) returned to normal glucose tolerance. Comparisons of prognoses between the two groups show that the rate of progression to diabetes in the quinoa group was significantly lower than in the control group (χ2 = 12.76, *p* = 0.002).

### Correlation analysis between different factors and diabetes

The lifestyle questionnaire variable data is shown in Table 5. The correlation analysis between various influencing factors and diabetes shows that quinoa consumption is significantly correlated with diabetes (*p* < 0.05). The analysis results are shown in Table 6.

**TABLE 5:** Variable assignment data from lifestyle questionnaire.

Characteristic	Assignment
gender^a^	Male = 1 Female = 2
age	Continuous variable
Family history of diabetes^a^	Yes = 1 No = 0
Edible quinoa^a^	Yes = 1 No = 0
Enjoy desserts or snacks^a^	Yes = 1 No = 0
Staple food structure^b^	equilibrium = 1
	Predominate of fine grain = 2
	Predominate of roughage = 3
	Hard to say = 4
Daily vegetable intake^b^	<100 g = 1
	100g - 250 g = 2
	250g - 500 g = 3
	>500 g = 4
Daily intake of water^b^	<3 cups = 1
	3-6 cups = 2
	6-9 cups = 3
	>9 cups = 4
Sugary drink intake^b^	Drink occasionally (1–2 times/week) = 1
	Drink regularly (3–5 times/week) = 2
	Drink every day (>5times/week) = 3
Frequency of exercise^b^	non-participation = 1
	Occasionally participation = 2
	Exercise 3 or more times per week on average, and for >30 min each time = 3
Daily sitting time^b^	<2 h = 1
	2–4 h = 2
	4–6 h = 3
	>6 h = 4
Daily sleep duration^b^	<5 h = 1
	5-7 h = 2
	7-9 h = 3
	>9 h = 4
Frequency of drinking^b^	Abstain from alcohol = 1 occasionally = 2
	frequently = 3
Smoking^a^	Yes = 1 No = 0

^a^ Binary variable.

^b^ Rank variable.

**TABLE 6:** Univariate analysis of different lifestyle factors and diabetes mellitus (*n* = 133).

Characteristic	Z/X^b^	*p*-Value
gender^a^	3.390	0.066
Age^b^	−0.155	0.877
Family history of diabetes^a^	0.206	0.650
Edible quinoa^a^	4.222	0.040
Enjoy desserts or snacks^a^	0.131	0.717
Staple food structure^b^	−0.869	0.385
Daily vegetable intake^b^	−0.160	0.873
Daily intake of water^b^	−0.364	0.716
Sugary drink intake^b^	−0.827	0.408
Frequency of exercise^b^	−1.302	0.193
Daily sitting time^b^	−1.178	0.239
Daily sleep duration^b^	−1.305	0.192
Frequency of drinking^b^	−0.990	0.322
Smoking^a^	0.909	0.340

^a^ Chi-square test.

^b^ Rank sum test.

### Logistical regression analysis of influencing factors of diabetes

At the end of 1 year of study, diabetes was taken as the dependent variable and gender, age, consumption of quinoa, the structure of staple food, intake of sugary drinks and exercise were taken as the independent variables (variable assignment shown in Table 4). Logistical regression analysis was conducted, and the results show that quinoa consumption as a dietary staple for 1 year is a statistically significant protective factor against delaying the progression of diabetes mellitus (*p* < 0.05), as shown in Table 7.

**TABLE 7:** Logistics Regression Analysis of different lifestyles factors and developing diabetes mellitus (n = 133).

	β	Standard error	Wald	*p*	Exp(β)	95% Confidence interval
gender	−1.207	.665	3.290	.070	.299	.081–1.100
age	−.005	.051	.009	.923	.995	.900–1.100
Family history of diabetes	.911	.669	1.857	.173	2.488	.671–9.225
Edible quinoa	−1.504	.653	5.299	.021	.222	.062–.800
Enjoy desserts or snacks	.051	.794	.004	.949	1.052	.222–4.994
Staple food structure	.206	.400	.264	.607	1.228	.561–2.692
Daily vegetable intake	.528	.517	1.043	.307	1.696	.615–4.675
Daily intake of water	−.007	.485	.000	.989	.993	.384–2.568
Sugary drink intake	.204	.559	.134	.715	1.227	.410–3.668
Frequency of exercise	−.911	.606	2.256	.133	.402	.123.-.1.320
Daily sitting time	−.590	.386	2.332	.127	.554	.260–1.182
Daily sleep duration	−1.032	.734	1.979	.160	.356	.085–1.501
Frequency of drinking	.263	.590	.199	.655	1.301	.410–4.134
Smoking	−.987	.899	1.204	.273	.373	.064–2.172
constant	5.691	4.723	1.452	.228	296.094	

## Discussion

In the past few decades, the prevalence of diabetes has increased significantly in almost all countries (Lovic et al., 2020), becoming a global public health problem. Pre-diabetes is a necessary clinical stage for the occurrence and development of diabetes. Epidemiological investigation (Tabák et al., 2012)shows that, without intervention, 5%–10% of people with impaired glucose tolerance will naturally develop type 2 diabetes every year. Therefore, early intervention can prevent the onset of diabetes or delay its progression in the pre-diabetic population. Dietary intervention is the fundamental treatment of diabetes and an essential intervention.

Quinoa has a highly comprehensive nutritional value, rich in protein, minerals, cellulose, vitamins and other elements. Compared with other common grains such as wheat, rice and millet, quinoa has lower starch content and higher soluble and insoluble fiber content (Repo-Carrasco-Valencia and Serna, 2011; Lamothe et al., 2015). Previous studies (Abellán Ruiz et al., 2017; Díaz-Rizzolo et al., 2022)have found that a quinoa diet can reduce body weight, body mass index, waist circumference and glycosylated hemoglobin levels in people with impaired glucose tolerance. Additionally, the current study also found that total cholesterol, LDL cholesterol, and systolic and diastolic blood pressure in the quinoa group were lower than before the quinoa dietary intervention, suggesting that in addition to reducing postprandial blood glucose, quinoa has benefits in lipid metabolism and blood pressure for people with impaired glucose tolerance. The current study differs from these two studies (Abellán Ruiz et al., 2017; Díaz-Rizzolo et al., 2022). First, the participant number in the current study is more extensive, and the age range is much broader. Secondly, the quinoa group consumed quinoa for a more extended period of time. Previous studies mainly focused on postprandial blood glucose and nutrient intake changes during the quinoa diet. The current study focused on systematically investigating the effects of quinoa on reducing glucose tolerance, abnormal lipid metabolism, abnormal blood pressure and disease prognosis in the pre-diabetic population for the first time.

A case-control group is part of the design of the current study. In the intervention group, adding an appropriate amount of quinoa into rice or white flour products with the staple food daily, while the control group did not add a staple food intervention. One year later, 2 hours postprandial blood glucose, glycosylated hemoglobin, insulin resistance index, total cholesterol, low-density lipoprotein cholesterol, body mass index, waist circumference, and systolic and diastolic blood pressure in the quinoa group were significantly lower than those in the control group (*p* < 0.05). The outcome infers that quinoa can effectively reduce postprandial blood glucose, blood pressure and improve lipid metabolism and insulin resistance in patients with impaired glucose tolerance. Logistic regression analysis of the lifestyle factors affecting diabetes showed that quinoa consumption is a protective factor for developing diabetes (*p* < 0.05).

Quinoa is considered a complete food, containing a variety of vitamins, minerals, unsaturated fatty acids, dietary fiber, rich protein and a balanced supply of essential amino acids (Lin M. et al., 2019), as well as being a rich source of bioactive phytochemicals such as polyphenols, flavonoids, rutin and saponins (Lin T. A. et al., 2019; Martínez-Villaluenga et al., 2020). Studies have reported that these phytochemicals in quinoa potentially benefit human health, reducing the risk of oxidative stress-related diseases such as cancer, cardiovascular disease, diabetes and obesity (Tang and Tsao, 2017). Some studies have also found that quinoa can significantly improve lipid accumulation and glucose homeostasis in the liver of obese mice as well as insulin resistance in animals with lipid metabolism disorder (Noratto et al., 2019; Selma-Gracia et al., 2021), and can repair impaired glucose tolerance in obese mice (An et al., 2021). The current study reports similar findings for the participants in this cohort. The prognoses of the two groups following dietary intervention were compared, and the conversion rate to diabetes in the quinoa group (7.8%) was significantly lower than that in the control group (20.3%). The conversion rate to normal (non-pre-diabetic) glucose tolerance in the quinoa group was 23.4%, significantly higher than 4.3% in the control group. These results suggest that quinoa can improve glucose tolerance and delay the progression of diabetes in patients with impaired glucose tolerance. This study appears to be the first report of quinoa affecting the prognosis and development of clinical Type 2 diabetes mellitus base on large-scale cohort study.

In a 10-year follow-up analysis of studies on metformin, the risk of diabetes progression was 18% lower in the metformin group and 34% lower in the lifestyle intervention group compared with the placebo (Knowler et al., 2009). Other studies have found that acarbose (Holman et al., 2017) and pioglitazone (Kernan et al., 2016) can reduce the risk of diabetes development by 18% and 52%, compared with a placebo. The current study found that quinoa consumption is associated with a 19.1% reduction in the risk of developing diabetes compared to the control group, comparable to the effect of drug interventions to delay the progression of diabetes. It has been found that lifestyle intervention can delay the onset of diabetes by 11 years, and metformin can only delay the onset of diabetes by 3 years (Herman et al., 2005), indicating that lifestyle intervention has greater benefits for people in a pre-diabetic phase. Quinoa is a readily available food and is more accepted by the population, making it suitable for promotion in the pre-diabetic population.

In addition to flavonoids (Tang and Tsao, 2017), the anti-diabetic effect of quinoa may correlate with reports that quinoa oil (11.9% of quinoa seeds) improves insulin sensitivity (Vega-Gálvez et al., 2010), leucine (0.08% of quinoa seeds) and other amino acids regulate the blood glucose and weight gain of animals (Zhang et al., 2007), and promote the secretion of insulin by pancreatic beta cells (Yang J. et al., 2010). The specific mechanism of quinoa in delaying the development of diabetes needs to be further studied.

A community-based cross-sectional study showed a strong association between blood lipids and type 2 diabetes and pre-diabetes (Bhowmik et al., 2018). Therefore, dyslipidemia in the pre-diabetic population also requires management and intervention. In the quinoa group data following the 1-year intervention, the total cholesterol and low-density lipoprotein cholesterol were significantly lower than at the start of the study, while the high-density lipoprotein cholesterol was significantly higher (*p* < 0.05). Triglyceride levels decreased after ingesting quinoa as a dietary staple, compared with those at the start of the study, but the difference was not statistically significant. A study (An et al., 2021) showed that quinoa intervention could reduce triglyceride levels, total cholesterol and LDL cholesterol, and upregulate HDL cholesterol levels. Another study reported that quinoa could effectively inhibit the increase of liver triglyceride and total cholesterol levels (Song et al., 2021) in high-fat-fed mice. However, Diana et al. found that 50 g of quinoa intake a day could reduce the serum triglycerides of overweight and obese participants, with no significant effect on total cholesterol, low-density lipoprotein and high-density lipoprotein cholesterol (*p* > 0.05) (Navarro-Perez et al., 2017). Song et al. (Song et al., 2021) found that low quinoa intake could regulate genes related to lipid metabolism, while high quinoa intake mainly regulated lipid metabolism and immune response genes. Given the differences in outcomes of the various studies, quinoa’s specific effect on lipid metabolism regulation needs further research.

However, some limitations should be acknowledged. This study is a single-center study, and the number of participants and study duration both need to be increased in further investigations.

The results of our study indicate that adding quinoa as a staple dietary food can regulate glucose tolerance and reduce blood glucose and lipid levels in people. Therefore, it is anticipated to improve the postprandial blood glucose of these people and change the development process of diabetes by adding an appropriate amount of quinoa into rice, and appropriately adjusting the staple food structure in the population with impaired glucose tolerance. Considering various factors in the occurrence and development of diabetes, more studies are required to further elucidate the molecular mechanism of quinoa on glycolipid metabolism.

## Data Availability

The original contributions presented in the study are included in the article/Supplementary material, further inquiries can be directed to the corresponding author.
